# Optimizing Machine Learning Algorithms for Landslide Susceptibility Mapping along the Karakoram Highway, Gilgit Baltistan, Pakistan: A Comparative Study of Baseline, Bayesian, and Metaheuristic Hyperparameter Optimization Techniques

**DOI:** 10.3390/s23156843

**Published:** 2023-08-01

**Authors:** Farkhanda Abbas, Feng Zhang, Muhammad Ismail, Garee Khan, Javed Iqbal, Abdulwahed Fahad Alrefaei, Mohammed Fahad Albeshr

**Affiliations:** 1 School of Computer Science, China University of Geosciences, Wuhan 430074, China; fengzhang@cug.edu.cn; 2 Department of Computer Science, Karakoram International University, Gilgit 15100, Pakistan; muhammad.ismail@kiu.edu.pk; 3 School of Geography, Karakoram International University, Gilgit 15100, Pakistan; garee.khan@kiu.edu.pk; 4 School of Environmental Studies, China University of Geosciences, Wuhan 430074, China; javediqbal@cug.edu.cn; 5 Department of Zoology, College of Science, King Saud University, P.O. Box 2455, Riyadh 11451, Saudi Arabia; afrefaei@ksu.edu.sa (A.F.A.); albeshr@ksu.edu.sa (M.F.A.)

**Keywords:** machine learning, optimization techniques, geospatial data, accuracy, scalability, practical implementation

## Abstract

Algorithms for machine learning have found extensive use in numerous fields and applications. One important aspect of effectively utilizing these algorithms is tuning the hyperparameters to match the specific task at hand. The selection and configuration of hyperparameters directly impact the performance of machine learning models. Achieving optimal hyperparameter settings often requires a deep understanding of the underlying models and the appropriate optimization techniques. While there are many automatic optimization techniques available, each with its own advantages and disadvantages, this article focuses on hyperparameter optimization for well-known machine learning models. It explores cutting-edge optimization methods such as metaheuristic algorithms, deep learning-based optimization, Bayesian optimization, and quantum optimization, and our paper focused mainly on metaheuristic and Bayesian optimization techniques and provides guidance on applying them to different machine learning algorithms. The article also presents real-world applications of hyperparameter optimization by conducting tests on spatial data collections for landslide susceptibility mapping. Based on the experiment’s results, both Bayesian optimization and metaheuristic algorithms showed promising performance compared to baseline algorithms. For instance, the metaheuristic algorithm boosted the random forest model’s overall accuracy by 5% and 3%, respectively, from baseline optimization methods GS and RS, and by 4% and 2% from baseline optimization methods GA and PSO. Additionally, for models like KNN and SVM, Bayesian methods with Gaussian processes had good results. When compared to the baseline algorithms RS and GS, the accuracy of the KNN model was enhanced by BO-TPE by 1% and 11%, respectively, and by BO-GP by 2% and 12%, respectively. For SVM, BO-TPE outperformed GS and RS by 6% in terms of performance, while BO-GP improved results by 5%. The paper thoroughly discusses the reasons behind the efficiency of these algorithms. By successfully identifying appropriate hyperparameter configurations, this research paper aims to assist researchers, spatial data analysts, and industrial users in developing machine learning models more effectively. The findings and insights provided in this paper can contribute to enhancing the performance and applicability of machine learning algorithms in various domains.

## 1. Introduction

Multiple fields of application, such as visual computing, language comprehension, suggestion engines, consumer activity analysis, and marketing, have widely applied machine learning (ML) algorithms on a massive scale [1]. This is owing to the reality that they are versatile and proficient at solving data diagnosing issues. Different ML algorithms are appropriate for diverse varieties of datasets and issues [2]. Overall, developing competent ML models necessitates efficient fine-tuning of hyperparameters based on the specifications of the chosen model [3].

Several alternatives must be examined to design and implement the most efficient ML model. Hyperparameter optimization is the method of crafting an ideal model architecture using the optimal hyperparameter configuration. The process of refining hyperparameters is deemed crucial in generating a thriving machine learning model, specifically for deep neural networks and tree-based ML models, which contain an abundance of hyperparameters. The hyperparameter optimization process differs across ML algorithms due to the varied kinds of hyperparameters they employ, such as discrete, categorical, and continuous hyperparameters [4]. The non-automatic traditional manual testing approach for hyperparameter tuning is still widely used by advance degree research students, despite the requirement for a thorough comprehension of ML algorithms and the importance of their hyperparameter configurations [5]. Nevertheless, because of various factors, including complex models, numerous hyperparameters, lengthy assessments, and non-linear hyperparameter relationships, manual tuning is not effective for several reasons. These factors have spurred additional research on techniques for automatic hyperparameter optimization, known as “hyper-parameter optimization” (HPO) [6].

The principal objective of hyperparameter optimization (HPO) is to streamline the hyperparameter tuning system and empower users to effectively implement machine learning models to address real-world problems [3]. Upon completion of an HPO procedure, one expects to obtain the optimal architecture for an ML model. Below are some noteworthy justifications for utilizing HPO techniques with ML models:

1. As numerous ML programmers devote significant time to adjusting the hyperparameters, notably for huge datasets or intricate ML algorithms having numerous hyperparameters, it decreases the degree of human labor required.
2. It boosts the efficacy of ML models. Numerous ML hyperparameters have diverse optimal values to attain the best results on different datasets or problems.
3. It boosts the replicability of the frameworks and techniques. Several ML algorithms may solely be justly assessed when the identical degree of hyperparameter adjustment is applied; consequently, utilizing the equivalent HPO approach to several ML algorithms also assists in recognizing the ideal ML model for a specific problem.

To identify the most appropriate hyperparameters, selecting the appropriate optimization technique is necessary. As a considerable number of HPO problems are complex nonlinear optimization challenges, they might not lead to a global optimum but rather to a local one. Therefore, standard optimization methods are possibly inappropriate for HPO issues [7]. For continuous hyperparameters, the gradients can be computed by means of gradient descent-based techniques, which are a typical variant of conventional optimization algorithms [8]. As an example, a gradient-based method may be employed to enhance the learning rate in a neural network.

Numerous other enhancement methods, like decision-theoretic techniques, multi-fidelity optimization methods and Bayesian optimization models, and metaheuristic algorithms, are better suited for HPO challenges in contrast to traditional optimization techniques like gradient descent [4]. Several of these algorithms can precisely determine conditional, categorical, and discrete hyperparameters as well as continuous hyperparameters.

The methods based on decision theory are founded on the idea of constructing a search space for hyperparameters, identifying the hyperparameter combinations within the search space, and choosing the combination of hyperparameters with the highest performance. A decision-theoretic strategy called grid search (GS) [9] involves scanning through a predetermined range of hyperparameter values. Random search (RS) [10], another decision-theoretic approach, is used when execution time and resources are limited, and it randomly selects hyperparameter combinations from the search space. In GS and RS, each hyperparameter configuration is verified individually.

Bayesian optimization (BO) [11] models, in contrast to GS and RS, deduce the subsequent hyperparameter value derived from the outcomes of the tried hyperparameter values, avoiding several unnecessary assessments. Consequently, BO can recognize the optimal hyperparameter fusion with fewer rounds of testing than GS and RS. BO can employ multiple models like the tree-structured Parzen estimators (TPEs), the random forest (RF), and the Gaussian process (GP) [12]. As a surrogate function to model the distribution of the objective function for various scenarios, BO-RF and BO-TPE [12] can preserve the dependency of factors. Conditional hyperparameters, such as the kernel type and A support vector machine’s (SVM) punishment parameter C, can be optimized using them. Parallelizing BO models is demanding because they function sequentially to strike a balance between discovering unexplored areas and exploiting regions that have already been tested.

Training an ML model often demands extensive labor and resources. To address resource constraints, multi-fidelity optimization algorithms, particularly those based on bandits, are widely used. A prevalent bandit-based optimization method called Hyperband [13] is an advanced version of RS. It produces downsized datasets and assigns an equal budget to every cluster of hyperparameters. To save time and resources, Hyperband discards inferior hyperparameter configurations in each cycle.

HPO problems are classified as intricate, non-linear, and extensive search space optimization problems, which are tackled utilizing metaheuristic algorithms [14]. The two most commonly employed metaheuristic algorithms for HPO are Particle Swarm Optimization (PSO) and Genetic Algorithm (GA) [15,16]. In each iteration, genetic algorithms determine the most optimal hyperparameter fusion and transmit those combinations to the ensuing iteration. In each cycle, every particle in PSO algorithms interacts with other elements to identify and revise the present global peak until it reaches the ultimate peak. Metaheuristics can efficiently explore the area and discover optimal or almost optimal solutions. Because of their superior efficiency, they are highly appropriate for HPO problems with extensive arrangement spaces.

Despite the fact that HPO algorithms are immensely useful in refining the effectiveness of ML models by adjusting the hyperparameters, other factors, such as their computational intricacy, still have a lot of room for progress. However, as different HPO models have distinct advantages and limitations that make them suitable for addressing specific ML model types and issues, it is vital to take them all into account when selecting an optimization algorithm. This academic article provides the subsequent contributions:

1. It encompasses three well-known machine learning algorithms (SVM, RF, and KNN) and their fundamental hyperparameters.
2. It assesses conventional HPO methodologies, their pros and cons, to facilitate their application to different ML models by selecting the fitting algorithm in pragmatic circumstances.
3. It investigates the impact of HPO techniques on the comprehensive precision of landslide susceptibility mapping.
4. It contrasts the increase in precision from the starting point and predetermined parameters to fine-tuned parameters and their impact on three renowned machine learning methods.

This overview article provides a comprehensive analysis of optimization approaches used for ML hyperparameter adjustment issues. We specifically focus on the application of multiple optimization approaches to enhance model accuracy for landslide susceptibility mapping. By optimizing the hyperparameters of these models, their performance can be significantly improved. Fine-tuning the hyperparameters allows the models to better capture the complex relationships between the input factors and landslide occurrences, resulting in more accurate and reliable predictions [17]. Landslide susceptibility mapping often covers large areas and requires the processing of extensive geospatial datasets. Optimizing the hyperparameters helps in achieving efficient resource allocation, such as computational power and memory usage, which is crucial for handling such data-intensive tasks. Efficient models can handle large datasets and expedite the mapping process, making it more practical for real-time or near-real-time applications [18,19]. Landslide susceptibility can vary across different geographic locations due to variations in geological, topographical, and environmental conditions. Optimizing the hyperparameters of the ML models ensures that the models can adapt and generalize well to different locations. This adaptability allows the models to be applied to new areas without extensive manual re-tuning of the hyperparameters, making the mapping process more scalable and efficient [20,21]. Our discussion encompasses the essential hyperparameters of well-known ML models that require optimization, and we delve into the fundamental principles of mathematical optimization and hyperparameter optimization. Furthermore, we examine various advanced optimization techniques proposed for addressing HPO problems. Through evaluation, we assess the effectiveness of different HPO techniques and their suitability for ML algorithms such as SVM, KNN, and RF.

To demonstrate the practical implications, we present the outcomes of applying various HPO techniques to three machine learning algorithms (SVM, KNN, and RF). We thoroughly analyze these results and also provide experimental findings from the application of HPO on a landslide dataset. This allows us to compare different HPO methods and explore their efficacy in realistic scenarios. In conclusion, this overview article provides valuable insights into the optimization of hyperparameters in machine learning, offering guidance for researchers and practitioners in selecting appropriate optimization techniques and effectively applying them to enhance the performance of ML models in various applications. The article tries to highlight the significance of hyperparameter tuning in machine learning models and the impact it has on model performance. It emphasizes the need for efficient HPO techniques and explores various optimization methods suitable for different types of hyperparameters. The information categorizes machine learning algorithms based on the characteristics of their hyperparameters, such as discrete, continuous, conditional, and categorical. It demonstrates how understanding these categories can guide the selection of appropriate HPO methods for optimizing hyperparameters in different ML models.

### Study Area

**Figure 1:**
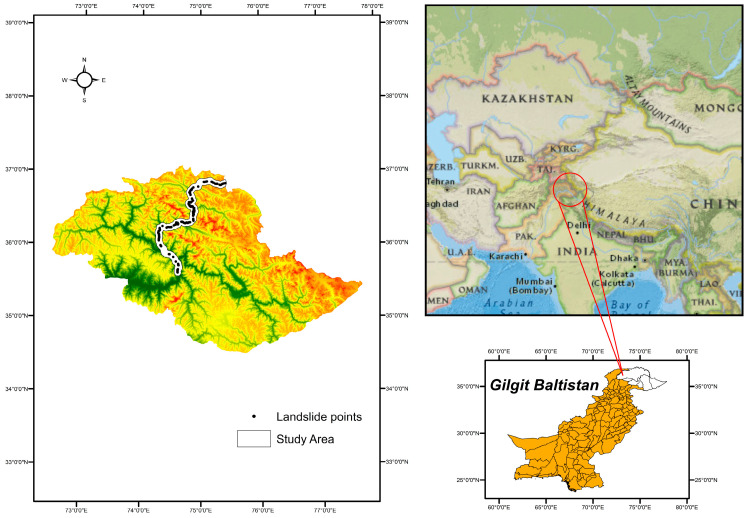
Study area for our experiment.

A region of roughly 332 km of the KKH expressway was analyzed. Conversely, the entire expanse of the route amounts to 1300 km, joining different provinces of Pakistan, like Punjab, Khyber Pakhtunkhwa, and Gilgit Baltistan with Xinjiang, an independent territory of China. The analysis was conducted in the north of Pakistan in the Gilgit, Hunza, and Nagar districts. There are various settlements along the KKH from Juglot, situated between 36°12′147″ N latitude and 74°18′772″ E longitude, moving through Jutal, Rahimbad, Aliabad, and culminating at Khunjarab Top, the China–Pakistan border crossing. The locality is positioned along the Indus River, Hunza River, and Gilgit River [22]. The evaluated zone measures 332 km in length and 10 km in radius, covering 3320 km^2^ along the KKH. The majority of the area is hilly, with the highest peak reaching 5370 m and the lowest elevation being 1210 m. Snowslides, mudslides, and tremors are frequent natural hazards in this region [23]. A rockslide or rubble fall set off by precipitation or seismic movements is the most prevalent type of landslide in our evaluation domain (Figure 1).

## 2. Methodology

As a starting point, the landslide dataset along KKH, which is a pure classification problem, serves as the gauge dataset for the examination of the HPO method on the data analysis issue.

The subsequent step involves configuring the ML models with their objective function. Based on the characteristics of their hyperparameters, all popular ML models are categorized into five groups, explained in Section 3. The three most common examples of these ML categories are “one categorical hyper-parameter”, “a few conditional hyper-parameters”, and “a wide hyper-parameter configuration space with multiple categories of hyper-parameters” [6,24,25,26]. RF, KNN, and SVM are chosen as the three ML algorithms to be adjusted since their hyperparameter types correspond to the three typical HPO scenarios. Each sample’s closest neighbor in terms of KNN is a crucial hyperparameter; the penalty parameter and the kernel type C are a few conditional hyperparameters in SVM. As described in Section 6, RF has many kinds of hyperparameters. Additionally, KNN, SVM, and RF can solve classification problems [27,28,29,30,31].

The evaluation metric and evaluation technique are determined in the subsequent step. The HPO methods employed in our experiment on the chosen dataset are evaluated using 3-fold cross-validation. In our experiments, the two most common performance measurements are utilized. The accuracy, which is the ratio of precisely labeled data, is used as the classifier performance parameter for classification models, and the model efficiency is also calculated using the computational time (CT), which is the overall time required to complete an HPO procedure with threefold cross-validation [32,33].

Subsequently, a number of criteria must be met to accurately compare various optimization methods and frameworks. In order to compare different HPO techniques, we first utilize the same hyperparameter configuration space. For each evaluation of an optimization approach, the single hyperparameter for KNN, ‘n neighbors’, is set to be in a similar span of 1 to 20. For each type of problem, the hyperparameters for SVM and RF models for classification problems are also set to be in the same configuration space. Table 1 displays the characteristics of the setup space for ML models.

Drawing from the notions presented in Section 3 and manual experimentation, the selected hyperparameters and their exploration domain are identified. Table 1 likewise details the hyperparameter categories for each ML technique.

**Figure 2:**
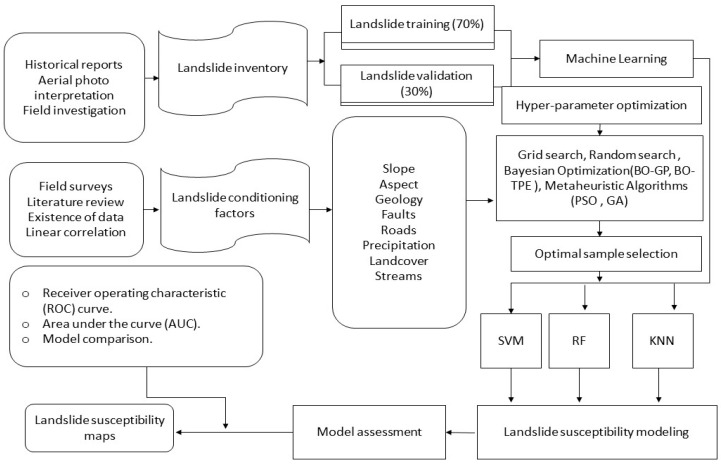
Methodology for hyperparameter selection for different ML algorithms for landslide susceptibility mapping.

Section 4 introduces six different hyperparameter optimization (HPO) approaches. To evaluate their performance, we chose six representative HPO methods discussed in Section 4, namely Grid Search (GS), Genetic Algorithm (GA), Random Search (RS), Bayesian Optimization with Gaussian Process (BO-GP), Bayesian Optimization with Tree-structured Parzen Estimator (BO-TPE), and Particle Swarm Optimization (PSO). To ensure unbiased empirical conditions for each HPO approach, the HPO experiments were carried out based on the procedures outlined in Section 2. Python 3.5 was used for all experiments, which were carried out on a system with a Core i7 processor and 32 GB of RAM. To investigate the associated machine learning and HPO methods, a variety of open-source Python modules and frameworks were used, encompassing sklearn [34], Skopt [35] Hyperopt [36], Optunity [37], Hyperband [13], BOHB [38], and TPOT [39]. The Figure 2 explain the methodology used for hyperparameter optimization for landslide susceptibility mapping. 

### Landslide Conditioning Factors

In our research area (Figure 1), landslides are influenced by various factors that can be classified into four categories: topological, hydrological, geological, and anthropological.

Topological factors are related to the terrain characteristics and include slope and aspect. Slope angle is considered the primary variable for slope stability, while aspect-related variables like exposure to sunlight, winds, rainfall, soil moisture, and cracks can influence the frequency of landslides.

To assess slope and aspect, we utilized the SRTM DEM with a 30 m resolution. The slope angle map was divided into five classes based on the source [40], and the terrain aspect was divided into nine classes to study its effect on landslide occurrence.

Geological factors are crucial in understanding landslide susceptibility, since different geological units have varying susceptibilities to geomorphological processes [41]. For this investigation, we considered geology and closeness to faults. We used the geological map of Pakistan to digitize fault lines and identify thirteen geological formations (classes) listed in Table 2.

Hydrological parameters, namely precipitation and proximity to streams, were also taken into account, since rainfall and water erosion are frequent triggers for landslides in this study location.

Anthropological factors encompass land use and distance to highways. We produced a land cover map using Sentinel 2 images and supervised classification, dividing the land cover into eight types to analyze its impact on landslide movement (Table 2). The accuracy of the land cover map was 87%, validated through a confusion matrix of LULC classification and field data.

Land use plays a significant role in landslide incidence, where barren slopes are more susceptible, while vegetative areas can help mitigate the occurrence of landslides [42,43]. Additionally, road development and construction activity can influence slope stability. The road network map was created through digitization from the Sentinel 2 image.

**Figure 3:**
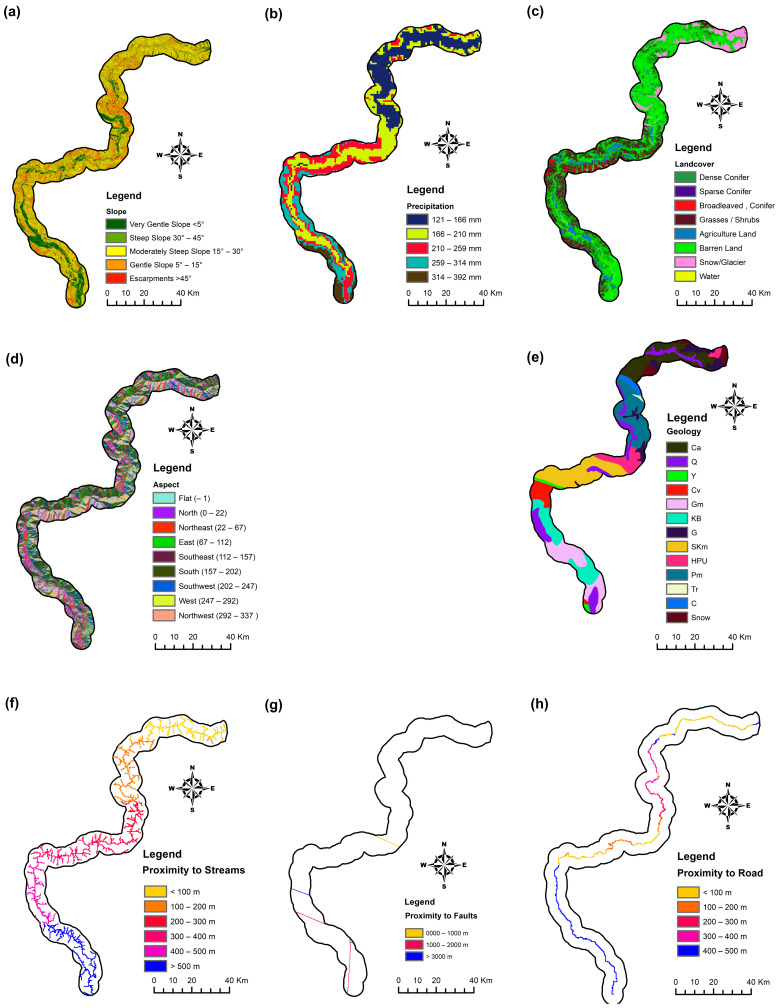
(**a**) Slope; (**b**) precipitation; (**c**) land cover; (**d**) aspect; (**e**) geology; (**f**) proximity to streams; (**g**) proximity to faults; (**h**) proximity to road.

In summary, our analysis considered eight factors for this case study: slope, aspect, land cover, geology, precipitation, distance to faults, distance to streams, and distance to roads, as depicted in Figure 3. These factors collectively contribute to the understanding of landslide occurrence in our research area (Table 2).

## 3. Hyperparameters

Hyperparameter configuration characteristics can be used to categorize ML algorithms. Based on these features, suitable optimization methods can be selected to optimize the hyperparameters.

### 3.1. Discrete Hyperparameter

A discrete hyperparameter typically needs to be modified for some ML algorithms, such as specific neighbor-based, clustering, and dimensionality reduction algorithms. The primary hyperparameter for KNN is the number of considered neighbors, or k. The number of clusters is the most important hyperparameter for k-means, hierarchical clustering, and EM. Similar to this, the fundamental hyperparameter for dimensionality reduction techniques like PCA and LDA is “n components,” or the quantity of features to be retrieved. The best option under these circumstances is Bayesian optimization, and the three surrogates might be evaluated to see which is most effective. Another excellent option is Hyperband, which may have a quick execution time because of its parallelization capabilities. In some circumstances, users may want to fine-tune the ML model by taking into account other less significant hyperparameters, such as the distance metric of KNN and the SVD solver type of PCA; in these circumstances, BO-TPE, GA, or PSO could be used [9,25].

### 3.2. Continuous Hyperparameter

Several naive Bayes algorithms, such as multinomial NB, Bernoulli NB, and complement NB, as well as several ridge and lasso methods for linear models, typically only have one crucial continuous hyperparameter that needs to be set. The continuous hyperparameter for the ridge and lasso algorithms is “alpha,” or the regularization strength. The key hyperparameter, commonly known as “alpha,” in the three NB algorithms stated above really refers to the additive (Laplace/Lidstone) smoothing value. The best option among these ML algorithms is BO-GP since it excels at optimizing a constrained set of continuous hyperparameters. Although gradient-based algorithms are also possible, they may only be able to find local optimum locations, making them less efficient than BO-GP [9,25,44].

### 3.3. Conditional Hyperparameters

It is apparent that many ML algorithms, including SVM, LR, and DBSCAN, have conditional hyperparameters. ‘penalty’, ‘C’, and the solver type are the three correlated hyperparameters of LR. Similar to DBSCAN, ‘eps’ and ‘min samples’ need to be tweaked together. SVM is more complicated because, after choosing a new kernel type, a unique set of conditional hyperparameters must be calibrated. As a result, some HPO techniques, such as GS, RS, BO-GP, and Hyperband, which cannot successfully optimize conditional hyperparameters, are not appropriate for ML models with conditional hyperparameters. If the correlations between the hyperparameters are known in advance, BO-TPE is the ideal option for these ML approaches. SMAC is an additional option that works well for fine-tuning conditional hyperparameters. You can also utilize GA and PSO [26,45].

### 3.4. Categorical Hyperparameters

Given that their primary hyperparameter is a categorical hyperparameter, ensemble learning algorithms tend to use this category of hyperparameter. The categorical hyperparameter for bagging and AdaBoost is “base estimator,” which is configured to be a single ML model. ‘Estimators’ is the term used for voting and denotes a list of ML single models that will be integrated. ‘Voting’ is a further categorical hyperparameter of the voting method that is used to select between a hard and soft voting approach. To evaluate whether these categorical hyperparameters are a viable base for machine learning, GS would be adequate. However, other hyperparameters, such as ‘n estimators’, ‘max samples’, and ‘max features’ in bagging, as well as ‘n estimators’ and ‘learning rate’ in AdaBoost, frequently need to be taken into account; as a result, BO algorithms would be a better option to optimize these continuous or discrete hyperparameters. In conclusion, the most appropriate HPO method should be chosen based on the characteristics of its hyperparameters when adjusting an ML model to obtain high model performance and low computing costs [9,46].

### 3.5. Big Hyperparameter Configuration Space with Different Types of Hyperparameters

Since they have numerous hyperparameters of diverse, different types, tree-based algorithms in ML, such as DT, RF, ET, and XGBoost, as well as DL algorithms, such as DNN, CNN, and RNN, are the most difficult to fine-tune. PSO is the ideal option for these ML models since it allows for parallel executions to increase efficiency, especially for DL models that frequently require a significant amount of training time. Other techniques like GA, BO-TPE, and SMAC can also be utilized; however, they might take longer than PSO to complete because it is challenging to parallelize these approaches [46].

## 4. Hyperparameter Optimization Techniques

### 4.1. Babysitting

A primary method for adjusting hyperparameters is babysitting, often referred to as “Trial and Error” or graduate student descent (GSD) [5]. Pupils and intellectuals equally frequently utilize this completely hands-on adjustment technique. The procedure is simple: following the creation of a machine learning (ML) prototype, the student experiments with a range of potential hyperparameter values founded on knowledge, speculation, or examination of outcomes from prior evaluations. This procedure is reiterated until the student exhausts the time limit (often meeting a deadline) or is content with the outcomes. Consequently, this technique necessitates sufficient background knowledge and expertise to quickly find the optimal hyperparameter values. Because of various reasons, including numerous hyperparameters, intricate models, time-consuming prototype evaluations, and nonlinear hyperparameter interactions, manual adjustment is often unfeasible [6]. These concerns prompted further research into methods for self-regulating hyperparameter optimization [47].

### 4.2. Grid Search

A frequently utilized method for exploring the hyperparameter configuration space is grid search (GS) [2]. GS can be seen as a brute force approach that analyzes every feasible permutation of hyperparameters that is given to the matrix of configurations. When a user-defined bounded range of values is utilized, GS assesses the cross-product of those values [7].

GS is incapable of completely employing the fruitful zones alone. To discover the global maximum, the following procedure needs to be executed manually [2]:

1. Commence with a wide exploration region and sizable stride length.
2. Utilizing prior effective hyperparameter settings, diminish the exploration area and stride length.
3. Persist in repeating step 2 until the optimum outcome is achieved.

Grid search (GS) is easy to parallelize and deploy. Nonetheless, its primary drawback is that it becomes inefficient for hyperparameter configuration spaces with a high number of dimensions since it necessitates exponentially more evaluations as the number of hyperparameters increases. This exponential increase is known as the “curse of dimensionality” [37]. If GS involves k variables, and each one has n unique values, then the computational intricacy of GS grows exponentially at a rate of $O\left(n^{k}\right)$ [16]. Consequently, GS can only serve as a practical HPO method when the hyperparameter search space is limited.

### 4.3. Random Search

To surmount some of the limitations of grid search (GS), random search (RS) was introduced in [10]. RS, similar to GS, selects a specific sample quantity from the search space within upper and lower thresholds as potential hyperparameter values at random, trains these possibilities, and iterates the process until the cost limit is depleted. The RS hypothesis suggests that the global optima, or at least their close values, can be uncovered if the configuration space is sufficiently extensive. In spite of possessing a limited budget, RS is capable of exploring more terrain than GS [10].

Due to its autonomous nature, one of the fundamental benefits of Random Search (RS) is that it can be easily parallelized and resource allocation can be managed efficiently. Unlike Grid Search (GS), RS chooses a fixed number of parameter variations from the provided distribution, thereby increasing the operational efficiency by reducing the likelihood of idle periods on a small, insignificant search space.

The computational intricacy of Random Search (RS) is *O*(*n*), as the total number of assessments in RS is predetermined to be n prior to the optimization commencings [48]. Additionally, with enough h resources, RS can determine the global optimum or the nearly optimal solution [49].

Random search (RS) is more efficient than grid search (GS) for extensive search areas. However, because it disregards previously successful regions, there are still a significant number of unnecessary function evaluations [2].

Consequently, both RS and GS waste substantial amounts of time evaluating areas of the search space that perform poorly, as each iteration’s review is independent of prior evaluations. Other optimization methods, such as Bayesian optimization, which rely on information from past evaluations to guide subsequent evaluations, can overcome this issue [11].

#### Bayesian Optimization

Iterative Bayesian optimization (BO) is a common approach to solving HPO problems [50]. Contrasting GS and RS, BO uses past results to determine future evaluation points. BO employs two essential elements, a surrogate model and an acquisition function, to define the next hyperparameter configuration [51].

All sample points are aimed at corresponding to the surrogate model for the objective function. The optimization function utilizes the stochastic surrogate model’s prediction distribution to weigh the compromise between searching and manipulation and identify which points to use. Exploitation involves sampling in present areas where, according to the posterior distribution, the global optimum is most likely to occur, whereas exploration entails sampling in uncharted territory. BO models integrate the exploration and exploitation procedures to determine the best places and avoid missing improved configurations in uncharted territory [52].

The following are the essential stages of Bayesian optimization (BO) [11]:

1. Create a surrogate probabilistic model of the target function.
2. Find the best hyperparameter values on the surrogate model.
3. Employ these hyperparameter values to the existing target function for evaluation.
4. Add the most recent observations to the surrogate model.
5. Repeat steps 2 through 4 until the allotted number of iterative cycles is reached.

Therefore, BO operates by revising the surrogate model after each assessment of the target function. Because it can identify the optimal hyperparameter combinations by evaluating the results of prior tests and because operating a surrogate model is commonly much less expensive than running the actual target function, BO is more effective than grid search (GS) and random search (RS).

Although Bayesian optimization models are sequential techniques that are hard to parallelize, they can frequently identify nearly optimal hyperparameter combinations in a limited number of iterations [4].

Tree Parzen estimator (TPE), Gaussian process (GP), and random forest (RF) are commonly used surrogate models for BO. Derived from their surrogate models, the three primary categories of BO algorithms are BO-GP, BO-RF, and BO-TPE. In our study on landslide data, we used BO-GP and BO-TPE. Sequential model-based algorithm configuration (SMAC) is another name for BO-RF [53].

### 4.4. BO-GP

The Gaussian process (GP) is a widely used alternative model for objective function modeling in Bayesian optimization (BO) [50]. The predictions follow a normal distribution when the function f is a realization of a GP with a mean μ and a covariance $\sigma^{2}$ [54]:

(1) $$p\left(y|x,D\right)=N\left(y|\hat{\mu },{\hat{\alpha }}^{2}\right),\;\;\;\;\;$$

Let *D* denote the hyperparameter configuration space, where *y* = *f*(*x*) represents the outcome analysis for every hyperparameter *x*. Subsequent evaluation points are chosen from the precise bounds created by the BO-GP model after making predictions. The sample records are revised with every new data point tested, and the BO-GP model is reconstructed using the revised data. This process is iterated until completion.

BO-GP’s application to a dataset of size n has a time and space complexity of $O\left(n^{3}\right)$ and $O(n^{2})$, respectively [26]. The limitation of the number of instances to cubic complexity hinders its ability to be parallelized, which is a major disadvantage of BO-GP [3]. Moreover, it is mainly utilized for optimizing continuous variables.

### 4.5. BO-TPE

An alternative common surrogate model for Bayesian optimization (BO) is the TPE, a Parzen estimator based on a tree structure [9]. Instead of using the predictive distribution employed in BOGP [3], BO-TPE constructs two solidity functions, namely $l\left(x\right)$ and $g\left(x\right)$, to act as generative models for the entire variable range. The recorded results are divided into favorable and unfavorable outcomes based on a pre-defined percentile value $y^{*}$, and TPE is applied by utilizing simple Parzen windows [9] to model each group of results:

(2) $$p\left(x|y,\;D\right)=\left\{\begin{array}{c} l\left(x\right),\;\;\;\;\;\;\;\;\;\;\;\;\;\;\;\;\;\;\;\;\;\;\;\;if\;y<y^{*}\\ g\left(x\right),\;\;\;\;\;\;\;\;\;\;\;\;\;\;\;\;\;\;\;\;\;\;if\;y>y^{*} \end{array}\right.$$

The proportion between the two solidity functions is subsequently used to establish the fresh setups for assessment, mirroring the predicted enhancement in the acquisition function. The provided contingent interdependencies are conserved as the Parzen estimators are organized in a hierarchical format. Consequently, TPE inherently facilitates conditional hyperparameters [54]. BO-TPE has a lower time complexity of $O\left(nlogn\right)$ [3] compared to BO-GP.

BO techniques work well for many HPO issues, regardless of how uncertain, non-linear, or non-smooth the objective function f is. If BO models do not strike a balance between exploitation and exploration, they may only reach a local rather than a global optimum. RS is not limited by this disadvantage as it lacks a specific focus area. Additionally, BO approaches are difficult to parallelize as their intermediate results are interdependent [4].

### 4.6. Metaheuristic Algorithms

A category of algorithms recognized as metaheuristic algorithms [55] are often utilized for optimization problems. They are mainly stimulated by biological concepts. Metaheuristics can effectively address optimization problems that are not convex, not smooth, or not continuous, divergent from several traditional optimization methods. One of the primary classifications of metaheuristic algorithms is population-based optimization algorithms (POAs), which also involve evolutionary algorithms, genetic algorithms (GAs), particle swarm optimization (PSO), and evolutionary strategies. Each generation in a POA commences with the formation and modernization of a community; afterward, each person is evaluated until the global best is found [11]. The methods employed to form and choose populations are the fundamental differences among diverse POAs [14]. Since a given population of N individuals can be assessed on at most N threads or processors concurrently, POAs are effortless to parallelize [49]. The two most commonly utilized POAs for HPO problems are particle swarm optimization and genetic algorithms.

### 4.7. Genetic Algorithm (GA)

Among the most well-liked metaheuristic algorithms, the genetic algorithm (GA) [15] is grounded on the evolutionary concept that individuals with the best survival and environmental adaptability are more prone to endure and transfer those abilities to future generations. The ensuing generation might consist of both superior and inferior individuals, and they will also inherit the characteristics of their forebears. Superior individuals will have a higher chance of surviving and producing competent progeny, while the inferior individuals will gradually vanish from the population. The individual who is most adaptable will be acknowledged as the global optimum after several successions [56].

Each chromosome or individual functions as a hyperparameter when utilizing GA in HPO problems, and its decimal value functions as the hyperparameter’s genuine input value in each assessment. Each chromosome has a variety of genes, which are portrayed by binary digits. The genes on this precise chromosome are consequently subjected to crossover and mutation procedures. The population consists of of all values inside the initialized chromosome/parameter ranges, and the fitness function specifies the metrics used to evaluate the parameters [56].

As the optimal parameter values are often missing from the randomly initialized parameter values, it is crucial to perform multiple actions on the well-adapted chromosomes, involving crossover, selection, and mutation methods, to discover the best values [15]. Chromosome picking is performed by selecting those with high values in the fitness function. The chromosomes that have high fitness function values are predisposed to be inherited by the next generation, so they create new chromosomes with the superior attributes of their parents, in order to maintain a constant population size. Chromosome selection allows positive traits from one generation to be passed on to the following generations. Crossover is utilized to produce new chromosomes by transferring a segment of genes from various chromosomes. Mutation techniques can also be utilized to produce new chromosomes by randomly altering one or more chromosome genes. Techniques such as mutation and crossover promote diverse traits in later generations and decrease the chances of missing desirable traits [3].

The following are the primary GA procedures [55]:

1. Commence by randomly initializing the genes, chromosomes, and population that depict the whole exploration space, as well as the hyperparameters and their corresponding values.
2. Identify the fitness function, which embodies the main objective of an ML model, and employ the findings to evaluate each member of the current generation.
3. Use chromosome methodologies such as crossover, mutation, and selection to generate a new generation consisting of the subsequent hyperparameter values that will be evaluated.
4. Continue executing steps 2 and 3 until the termination criteria are met.
5. Conclude the process and output the optimal hyperparameter configuration.

Amidst the previously mentioned procedures, the population initiation phase is critical for both PSO and GA because it offers an initial approximation of the ideal values. A proficient population initiation approach can significantly accelerate convergence and enhance the effectiveness of POAs, despite the fact that the initiated values will be progressively enhanced throughout the optimization process. An appropriate starting population of hyperparameters ought not to be restricted to an unfavorable area of the exploration space and instead should include individuals in proximity to global optima by considering the encouraging domains [57].

In GA, random initiation, which merely generates the initial population with arbitrary values within the specified exploration space, is frequently utilized to produce hyperparameter configuration potential for the initial population [58]. As a result of its selection, crossover, and mutation operations, GA is uncomplicated to construct and does not necessitate exceptional initializations. This reduces the probability of losing out on the global optimum.

Consequently, it is beneficial to determine a probable acceptable initial exploration space for the hyperparameters when the data analyst has limited expertise. The primary disadvantage of GA is that the approach introduces novel hyperparameters that must be specified, such as the population magnitude, fitness function kind, crossover percentage, and mutation percentage. Additionally, GA is a successive execution method, making parallelization difficult. GA has an $O\left(n^{2}\right)$ time complexity [59]. GA may occasionally prove ineffective due to its slow convergence pace.

### 4.8. Particle Swarm Optimization (PSO)

A different group of evolutionary algorithms often utilized for optimization problems is the particle swarm optimization (PSO) [60]. PSO algorithms take inspiration from biological communities that demonstrate both individual and cooperative inclinations [14]. PSO functions by enabling a cluster of particles to navigate the exploration area in a partially random manner [6]. Through teamwork and exchange of knowledge among the particles within the cluster, PSO algorithms determine the optimal solution.

Collection S of n particles is present in PSO as [2]

(3) $$s=\left(s_{1}\;,\;s_{2}\;,\;\ldots \ldots \ldots ,\;s_{n}\right),\;$$

and every particle $S_{i}$ is expressed by a vector

$$S_{i}=\langle \vec{x_{i}}\;,\vec{\;v_{i}}\;,\vec{\;p_{i}}\rangle ,$$

where $\vec{x_{i}}\;$represents the current location, $\vec{\;v_{i}}\;$represents the current speed, and $\vec{\;p_{i}}$ is the best position of the swarm up until the current iteration.

Initially, every particle is generated randomly in terms of position and speed using PSO. Every particle then analyzes its current location and stores it along with its performance grades. In the subsequent iterations, the velocity of each particle $\vec{\;v_{i}}\;$is updated, rooted on its most recent global best position $\vec{p}$ and its previous position $\vec{\;p_{i}}$:

(4) $$\vec{\;v_{i}}≔\vec{\;v_{i}}+U\left(0,\varphi_{1}\right)\left(\vec{\;p_{i}}-\vec{\;x_{i}}\right)+U\left(0,\varphi_{2}\right)\left(\vec{p}-\vec{\;x_{i}}\right),$$

where the acceleration constants $\varphi_{1}$ and $\varphi_{2}\;$ are used to calculate the continuous uniform distributions $U\left(0,\varphi \right)$.

The particles then travel according to their new velocity vectors:

$$\vec{\;x_{i}}≔\vec{\;x_{i}}+\vec{\;v_{i}}$$

The above steps are iterated until the termination criteria are met in PSO. Contrasted to GA, PSO is simpler to execute because it does not require incremental steps like crossover and mutation. In GA, all chromosomes interact with each other, resulting in the entire population moving towards the optimal region uniformly. In contrast, PSO only shares knowledge on top individual particles and the global superior particles, resulting in a unidirectional transmission of information and the search process moving towards the current optimal solution [2]. The computational complexity of the PSO algorithm is O(nlogn) [61], and its convergence rate is generally faster than that of GA. Moreover, PSO particles function autonomously and are solely required to exchange information with each other after each iteration, making it easy to parallelize the process and increase model efficiency [6].

The primary deficiency of PSO is that it may solely attain a local rather than a global optimum, particularly for discrete hyperparameters, in the absence of appropriate population initialization [62]. Adequate population initialization can be achieved by developers with previous experience or population initialization methods. Multiple strategies for population inception, such as the opposition-based optimization algorithm [58] and the space transformation search approach [63], have been devised to enhance the performance of evolutionary algorithms. Additional population inception methods will require more resources and execution time. 

Table 3 provided the detailed comparisons between popular HPO techniques based on their time complexity and hyperparameter configuration characteristics can be used to categorize ML algorithms. Based on these features, suitable optimization methods can be selected to optimize the hyperparameters.

## 5. Mathematical and Hyperparameter Optimization

Machine learning is primarily used to address issues with efficiency. To accomplish this, a weight parameter improvement technique for an ML model is used until the objective function value reaches a minimum value and the accuracy rate reaches a maximum value. Similar to this, methods for optimizing hyperparameter configurations aim to improve a machine learning model’s architecture. The fundamental ideas of mathematical optimization are covered in this part, along with hyperparameter optimization for machine learning models.

### 5.1. Mathematical Optimization

The aim of mathematical optimization is to locate the optimal solution from a pool of possibilities of a maximized or minimized objective function [64]. Depending on whether restrictions are placed on the choice or the solution variables, optimization problems can be classified as either constrained or unconstrained. A decision variable *x* in unconstrained optimization problems can take on any value from the one-dimensional space of real numbers, *R*. This problem is an unconstrained optimization problem [65].

(5) $$\underset{x\in R}{\min}\;f\left(x\right)$$

where the goal function is *f(x)*.

In contrast, constrained optimization problems are more prevalent in real-world optimization problems. The decision variable *x* in constrained optimization problems must satisfy specific constraints, which can be equalities or inequalities in mathematics. Therefore, optimization problems can be expressed as general optimization problems or constrained optimization problems [65].

$$\underset{x}{\min}\;f\left(x\right)$$

subject to

(6) $$g_{i}\left(x\right)\leq 0,i=1,2,\ldots \ldots \ldots \ldots ,m$$

$$h_{j}\left(x\right)=0,j=1,2,\ldots \ldots \ldots \ldots ,p,$$

x ϵ X ,

where *X* is the domain of *x*, $g_{i}\left(x\right)\leq 0,i=1,2,\ldots \ldots \ldots \ldots ,m,\;$ are the inequality constraint functions, and $h_{j}\left(x\right)=0,j=1,2,\ldots \ldots \ldots \ldots ,p,$ are the equality constraint functions.

Constraints serve the purpose of limiting the feasible region, or the possible values of the optimal answer, to specific regions of the search space.

As a result, the feasible area *D* of *x* can be illustrated as follows:

(7) $$D=\left\{x\in X|g_{i}\left(x\right)\leq 0,h_{j}\left(x\right)=0\right\}.\;$$

An objective function *f*(*x*) that can be minimized or maximized, a collection of decision variables *x*, and an optimization problem are the three main components. The variables may be allowed to take on values within a certain range by a set of constraints that apply to the issue. if the optimization issue is constrained. Determining the collection of variable values that minimizes or maximizes the objective function while satisfying any necessary constraints is the aim of optimization problems.

The viable range of the cluster count in k-means, as well as temporal and spatial limitations, are typical constraints in HPO problems. Consequently, constrained optimization methods are frequently employed in HPO problems.

In many situations, optimization problems may converge to local optima rather than a global optimum. For example, when seeking the minimum value of a problem, suppose that D is the viable region of a decision factor x. A global minimum is the point $x^{*}\;\in D$ satisfying $f\left(x^{*}\right)\leq f\left(x\right)\forall x\in D$, whereas a local minimum is the point $x^{*}\in D$ in a vicinity N satisfying $f\left(x^{*}\right)\leq f\left(x\right)\forall x\in N\cap D$ [65]. As a result, the local optimum only exists in a limited range and might not be the best option for the full possible region.

Only convex functions have the guarantee that a local optimum is also the global optimum [66]. Convex functions are those that have a single optimum. Consequently, the global optimal value can be found by extending the search along the direction in which the objective function declines.

*f*(*x*) is a convex function if and only if [66], for $\forall x_{1},x_{2}\in X,\forall t\;\in \left[0,1\right],$

(8) $$f\left(tx_{1}+\left(1-t\right)x_{2}\right)\leq f\left(x_{1}\right)+\left(1-t\right)f\left(x_{2}\right),$$

where *t* is a coefficient with a range of [0, 1] and *X* is the domain of the choice variables. Only when the viable region C is a convex set and the objective function *f*(*x*) is a convex function is an optimization issue a convex optimization problem [66].

(9) $$\underset{x}{\min}\;f\left(x\right)\;$$

subject to x∈ C.

Conversely, nonconvex functions only have one global optimum while having several local optima. Nonconvex optimization problems make up the majority of ML and HPO issues. Inappropriate optimization techniques frequently only find local rather than global optima.

Traditional techniques such as Newton’s method, conjugate gradient, gradient descent, and heuristic optimization techniques can all be utilized to address optimization problems [64]. Gradient descent is a popular optimization technique that moves in the opposite direction of the positive gradient as the search trajectory approaches the optima. The global optimum, however, cannot be detected with certainty via gradient descent unless the objective function is convex. The Hessian matrix’s inverse matrix is used by Newton’s technique to determine the optimal solution. Despite needing more time and space to store and construct the Hessian matrix than gradient descent, Newton’s approach offers a faster convergence speed.

To find the best solution, conjugate gradient searches are conducted across a conjugated direction created by the gradient of known data samples. Conjugate gradient has a higher rate of convergence than gradient descent, but its computation is more difficult. Heuristic methods, in contrast to other conventional approaches, solve optimization issues by applying empirical rules rather than by following a set of predetermined processes to arrive at the solution. Heuristic techniques frequently find the estimated global optima after a few rounds, although they cannot always find the global optimum [64].

### 5.2. Hyperparameter Optimization

Throughout the ML model design phase, the optimal hyperparameters can be identified by efficiently exploring the hyperparameter space using optimization techniques [67,68]. The hyperparameter optimization procedure consists of four key constituents: an estimator, also known as a regressor or classifier with a goal function, a search space or configuration space, an optimization or search method to find combinations of hyperparameters, and an evaluation function to gauge how well different hyperparameter configurations work.

Hyperparameters, like whether to employ early halting or the learning rate, can have categorical, binary, discrete, continuous, or mixed domains. Thus, categorical, continuous, and discrete hyperparameters are the three categories of hyperparameters. The domains of continuous and discrete hyperparameters are often restricted in real-world applications. Hyperparameter configuration spaces can also include conditional hyperparameters, which must be adjusted based on another hyperparameter’s value [9,69]. In certain scenarios, hyperparameters have the flexibility to take on unrestricted real values, and the set of feasible hyperparameters, denoted as X, can be a vector space in n dimensions with real values. Nevertheless, in machine learning models, hyperparameters usually have specific value ranges and are subject to various constraints, which introduce complexity to their optimization problems as constrained optimization problems [70]. For instance, in decision trees, the number of features considered should vary from 0 to the number of features, and in k-means, the number of clusters should not exceed the data points’ size [7].

Moreover, categorical attributes typically possess a restricted range of allowable values, such as the activation function and optimizer choices in a neural network. Consequently, the complexity of the optimization problem is heightened because the feasible domain of hyperparameters, denoted as *X*, often exhibits a complex structure [70].

Typically, the goal of a hyperparameter optimization task is to obtain [16]

(10) $$x^{*}=arg\;\underset{x\in X}{\min}\;f\left(x\right)$$

A hyperparameter, denoted as *x*, is capable of assuming any value within the search space *X*. The objective function, *f*(*x*), which is to be minimized, could be the error rate or the root mean squared error (RMSE), for example. The optimal hyperparameter configuration, $x^{*}$, is the one that results in the best value of *f*(*x*).

The objective of HPO is to fine-tune hyperparameters within the allocated budgets to attain optimal or nearly optimal model performance. The mathematical expression of the function f varies depending on the performance metric function and the objective function of the chosen ML algorithm. Various metrics, such as F1-score, accuracy, RMSE, and false alarm rate, can be utilized to evaluate the model’s performance. In practical applications, time constraints must also be considered, as they are a significant limitation for optimizing HPO models. With a considerable number of hyperparameter configurations, optimizing the objective function of an ML model can be exceedingly time-consuming. Each time a hyperparameter value is assessed, the entire ML model must be retrained, and the validation set must be processed to produce a score that quantifies the model’s performance.

After choosing an ML algorithm, the primary HPO procedure involves the following steps [7]:

1. Choose the performance measurements and the objective function.
2. Identify the hyperparameters that need tuning, list their categories, and select the optimal optimization method.
3. Train the ML model using the default hyperparameter setup or common values for the baseline model.
4. Commence the optimization process with a broad search space, selected through manual testing and/or domain expertise, as the feasible hyperparameter domain.
5. If required, explore additional search spaces or narrow down the search space based on the regions where best-functioning hyperparameter values have been recently evaluated.
6. Finally, provide the hyperparameter configuration that exhibits the best performance.

The majority of typical optimization approaches [71] are inappropriate for HPO. However, HPO problems differ from conventional optimization methods in the following ways [7].

When it comes to HPO problems, conventional optimization techniques that are designed for convex or differentiable optimization problems are often not suitable due to the non-convex and non-differentiable nature of the objective function in ML models. Moreover, even some conventional derivative-free optimization methods perform poorly when the optimization target is not smooth [72].

ML models’ hyper-parameters contain continuous, discrete, categorical, and conditional hyperparameters, which means that numerous conventional numerical optimization techniques that only deal with numerical or continuous variables are not suitable for HPO problems [73].

In HPO approaches, computing an ML model on a large dataset can be costly, so data sampling is sometimes used to provide approximations of the objective function’s values. Therefore, efficient optimization methods for HPO problems must be capable of utilizing these approximations. However, many black-box optimization (BBO) methods do not consider the function evaluation time, which makes them unsuitable for HPO problems with constrained time and resource limits. To find the best hyperparameter configurations for ML models, appropriate optimization methods must be applied to HPO problems.

## 6. Hyperparameters in Machine Learning Models

### 6.1. KNN

The K-nearest neighbor (KNN) is a straightforward machine learning algorithm that classifies data samples based on their distance from one another. In a KNN, the forecasted category for each test point is determined by identifying the category that has the highest number of nearest neighbors in the training set, where the number of nearest neighbors is set to k.

Assuming that the training set $T=\left\{\left(x_{1},y_{1}\right),\left(x_{2},y_{2}\right),\ldots ,\left(x_{n},y_{n}\right)\right\}\;$, $x_{i}$ is the instance’s feature vector and $y_{i\;}\;\in \left\{C_{1},C_{2},C_{3},\ldots \ldots C_{m}\;\right\}$ is the class of the instance, while $i=\left(1,2,\ldots n\right)$, and the class y of a test instance x can be represented by

(11) $$y=arg\;\underset{cj}{\max}\underset{x_{i}\in N_{k}\left(x\right)}{\sum }I\left(y_{i}=c_{j}\right),\;i=1,2,\ldots .,n;j=1,2\ldots .,m,$$

$N_{k}\left(x\right)\;$is the field encompassing the k-nearest neighbors of *x*, and $I\left(x\right)$ is an indicator function with *I* = 1 when $y_{i}=c_{j}$ and *I* = 0 otherwise.

The primary hyperparameter in KNN is *k*, which determines the number of nearest neighbors to be considered [44]. If k is too small, the model may underfit the data, whereas if it is too large, the model may overfit the data and require significant computational resources. The choice of weighting function used for prediction can also affect the model’s performance, with “uniform” weighting treating all points equally, and “distance” weighting giving more weight to closer points. Additionally, the Minkowski metric can be improved by adjusting the distance metric and power parameter. The method used to find nearest neighbors can be selected from options such as a ball tree, a k-dimensional (KD) tree, or a brute force search. Setting the algorithm to “auto” in sklearn can allow the model to automatically select the most suitable algorithm [34].

### 6.2. SVM

A classification or regression problem can be addressed using a supervised learning technique called support vector machine (SVM) [74]. SVM algorithms operate on the premise that data points can be linearly separated by transforming them from a low-dimensional to a high-dimensional space and constructing a hyperplane as the classification boundary to separate the data samples [75]. In SVM, the objective function for n data points is given by [76]

(12) $$arg\;\underset{w}{\min}\left\{\frac{1}{n}\overset{n}{\underset{i=1}{\sum }}\max+cw^{T}w\right\},$$

The objective function for SVM with n data points involves a normalization vector w and a critical hyperparameter C, which also serves as the penalty parameter for the error term. The SVM model also allows for adjustment of the kernel function $f\left(x\right)$ used to compare two data points $x_{i}$ and $x_{j}$, with several kernel types available, including common kernel types as well as customized ones. Therefore, fine-tuning the kernel type hyperparameter is essential. Popular kernel types in SVM include linear, polynomial, radial basis function (RBF), and sigmoid kernels.

The various kernel operations can be represented as [77]

(13) $$\mathrm{Linear}\;\mathrm{kernel}:\;f\left(x\right)=x_{i}^{T}x_{j};$$

(14) $$\mathrm{Polynomial}\;\mathrm{Kernel}:\;f\left(x\right)={\left(\gamma x_{i}^{T}x_{j}+\gamma \right)}^{d};$$

(15) $$\mathrm{Sigmoid}\;\mathrm{kernel}:\;f\left(x\right)=\left(\tanh\left(x_{i}^{T}x_{j}+\gamma \right)\right)$$

(16) $$\mathrm{RBF}\;\mathrm{kernel}:\;f\left(x\right)=\exp\left(-\gamma {\left|\left|x-x^{\prime }\right|\right|}^{2}\right)$$

Once a kernel type is chosen, several other hyperparameters must be fine-tuned, as indicated in the equations for the kernel function. For kernel types such as polynomial, RBF, or sigmoid, the conditional hyperparameter is represented by ‘gamma’ in sklearn, while for polynomial and sigmoid kernels, it is γ, which can be specified using ‘coef0’ in sklearn. The polynomial kernel also has a conditional hyperparameter d that denotes the degree of the polynomial kernel function. Another hyperparameter in support vector regression (SVR) models is epsilon, which represents distance inaccuracy in the loss function [34].

### 6.3. Random Forest (Tree-Based Models)

The decision tree (DT) [78] is a prevalent classification technique that condenses a set of classification regulations from the data and applies them to a tree arrangement to delineate determinations and potential outcomes. A DT consists of three major parts: a root node that represents the whole data set, several decision nodes that represent decision tests and sub-node splits for each attribute, and numerous leaf nodes that represent the resultant classes [79]. To render accurate determinations on each subset, DT algorithms partition the training set iteratively into subsets with enhanced feature values. To avoid over-fitting, DT utilizes pruning, which involves discarding some of the sub-nodes of decision nodes. The maximal tree depth, or “max depth”, is an important hyperparameter of DT algorithms as a deeper tree encompasses more sub-trees to assist it in making more accurate inferences [80]. To fabricate efficient DT models, several other critical HPs must be fine-tuned [55]. Firstly, a measuring function, referred to as a “criterion” in Sklearn, can be established to determine the quality of splits. The two primary categories of measuring functions are Gini impurity and information gain [51]. The “splitter” split selection technique can optionally be altered to “best” or “random” to select the ideal split or a random split, respectively. Max features, the number of attributes taken into consideration to provide the optimal split, can also be tweaked as a feature selection procedure. Furthermore, to enhance performance, many discrete hyperparameters associated with the splitting procedure must be adjusted: the minimum number of data samples required to split a decision node or obtain a leaf node, designated by the terms “min samples split” and “min samples leaf,” respectively; the maximal number of leaf nodes and the minimal weighted fraction of the total weights, respectively, may also be tweaked to enhanced model performance [34,51]. Built on the idea of DT models, many decision-tree-based ensemble methods, such as random forest (RF), extra trees (ET), and extreme gradient boosting (XGBoost) models, have been created to enhance model performance by mixing several decision trees. In RF, elementary DTs are constructed on many randomly generated subsets, and the class with the majority vote is selected as the final classification outcome [81]. Another tree-based ensemble learning approach, ET, is analogous to RF in that it builds DTs from all samples and randomly selects the feature sets. Additionally, RF optimizes splits on DTs, while ET generates splits at random [82]. Tree-based ensemble models share the same hyperparameters as DT models in this subsection because they are established using decision trees as base learners. Apart from these hyperparameters, the number of decision trees to be combined—designated as “n estimators” in sklearn—must be adjusted for RF, ET, and XGBoost. There are several general ensemble learning techniques, besides tree-based ensemble models, that incorporate many individual ML models to produce superior model performance compared to any single algorithm alone. This article introduces three prevalent ensemble learning models: voting, bagging, and AdaBoost [83].

*The technique of ensemble learning known as voting* [83] is a basic approach that aggregates individual estimators to create a more precise and comprehensive estimator by implementing the majority voting principle. The voting mode in Sklearn can be modified from “hard” to “soft” to specify whether the final classification output will be determined by a majority vote or by averaging the predicted probabilities. It is also possible to modify the list of selected individual ML estimators and their corresponding weights in certain cases. For example, a more substantial weight can be assigned to a particular ML model that exhibits superior performance.

*Bootstrap aggregating* [83], also referred to as bagging, is an ensemble learning technique that creates a final predictor by training multiple base estimators on different randomly selected subsets. When using bagging methods, it is important to consider the type and number of base estimators in the ensemble, as indicated by the “base estimator” and “n estimators” parameters. Additionally, the “max samples” and “max features” parameters, which specify the sample and feature sizes to generate different subsets, can also be adjusted.

*AdaBoost* [83], which stands for adaptive boosting, is an ensemble learning technique that trains a sequence of base learners (weak learners), with later learners emphasizing the misclassified samples of earlier learners before training a final strong learner. This process involves retraining instances that were incorrectly classified with additional fresh instances and adjusting their weights so that subsequent classifiers focus more on challenging situations, gradually building a stronger classifier. The base estimator type in AdaBoost can be a decision tree or other techniques. In addition to these two hyperparameters, it is necessary to control the maximum number of estimators at which boosting is terminated, or “n estimators,” as well as the learning rate that reduces the contribution of each classifier’s estimators, in order to establish a trade-off between them.

## 7. Results

Table 4, Table 5 and Table 6 present the results of six different HPO methods applied to RF, SVM, and KNN classifiers on the landslide dataset. The default hyperparameter configurations of each model were used as the baseline, and then HPO algorithms were applied to assess their accuracy and computational time. The results show that default settings do not always lead to the best model performance, highlighting the importance of HPO techniques.

Among the baseline models for HPO, GS and RS were used, and the results indicate that GS often has significantly higher computational time than other optimization techniques. RF and SVM models are faster than GS, but neither of them can guarantee finding near-optimal hyperparameter configurations of ML models. BO and multi-fidelity models perform significantly better than GS and RS in terms of accuracy, but BO-GP often requires longer computation times due to its cubic time complexity.

BO-TPE and BOHB frequently perform better than other methods due to their ability to quickly compute optimal or almost optimal hyperparameter configurations. GA and PSO also frequently have higher accuracies than other HPO approaches for classification tasks. BO-TPE and PSO are often successful in finding good hyperparameter configurations for ML models with vast configuration spaces.

Overall, GS and RS are easy to implement but may struggle to find ideal hyperparameter configurations or take a long time to run. BO-GP and GA may take more time to compute than other HPO methods, but BO-GP performs better in small configuration spaces, while GA performs better in large configuration spaces. BO-TPE and PSO are effective for ML models with vast configuration spaces.

### 7.1. Landslide Susceptibility Maps

#### 7.1.1. Random Forest

**Figure 4:**
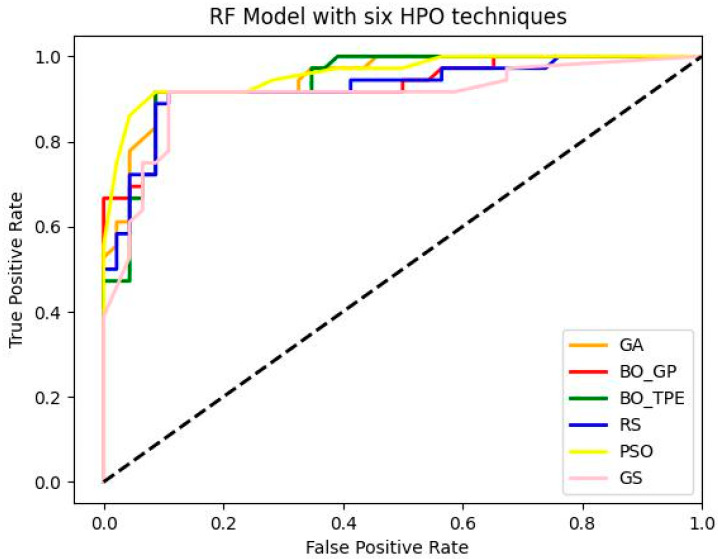
Receiver-operating characteristic (ROC) curve and AUC curve of Random forest (RF) model with GS (Grid Search), RS (Random Search), BO-GP (Bayesian optimization Gaussian process), BO-TPE (Bayesian optimization Tree-structured Parzen estimator), GA (Genetic Algorithm), and PSO (Particle Swarm Optimization) as parameter optimization techniques.

**Figure 5:**
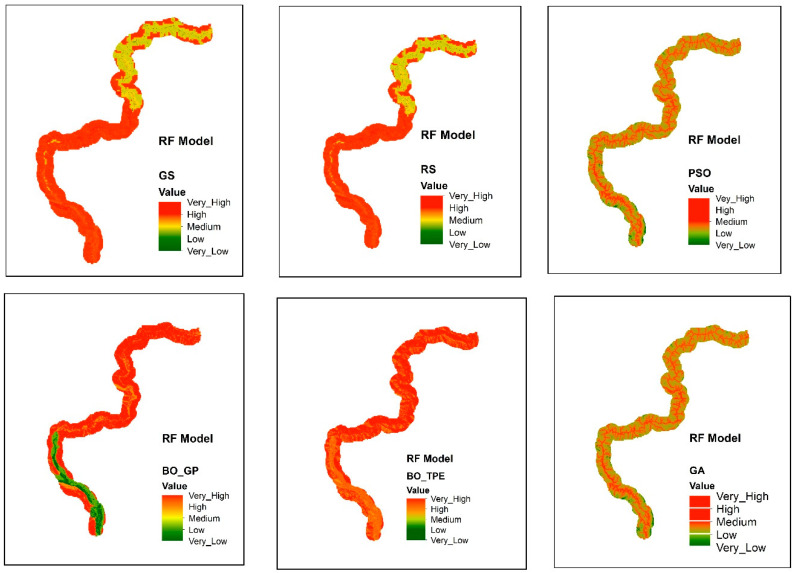
Landslide susceptibility maps obtained from Random Forest (RF) model using six different optimization techniques: GS (Grid Search), RS (Random Search), BO-GP (Bayesian optimization Gaussian process), BO-TPE (Bayesian optimization Tree-structured Parzen estimator), GA (Genetic Algorithm), and PSO (Particle Swarm Optimization).

The metaheuristic algorithms PSO and GA performed remarkably well, with PSO increasing accuracy from baseline optimization methods GS and RS by 5% and 3%, respectively, and GA increasing accuracy from baseline optimization techniques GS and RS by 4% and 2%. However, compared to GS and RS, the accuracy of the Bayesian optimization technique BO-TPE increased by 4% and 2%, respectively, and BO-GP by 3% and 1%. Thus, the overall accuracy of the RF model was increased via metaheuristic and Bayesian optimization, as shown in the Figure 4. As discussed earlier, the most challenging ML algorithms to optimize are tree-based algorithms like RF, because they have multiple hyperparameters of various different types. These ML models work best with PSO because it enables parallel executions, which boost productivity. Other methods like GA and BO-TPE can also be applied; however, they might take longer to finish than PSO does because it is difficult to parallelize these techniques. The susceptibility maps generated by RF are shown in Figure 5.

#### 7.1.2. KNN

**Figure 6:**
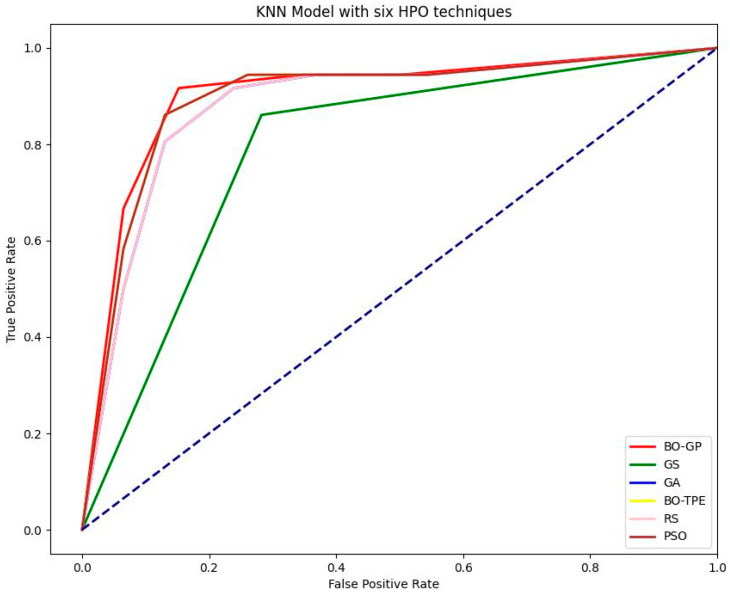
Receiver-operating characteristic (ROC) curve and AUC curve of K-nearest neighbors (KNN) model with GS (Grid Search), RS (Random Search), BO-GP (Bayesian optimization Gaussian process), BO-TPE (Bayesian optimization Tree-structured Parzen estimator), GA (Genetic Algorithm), and PSO (Particle Swarm Optimization) as parameter optimization techniques.

**Figure 7:**
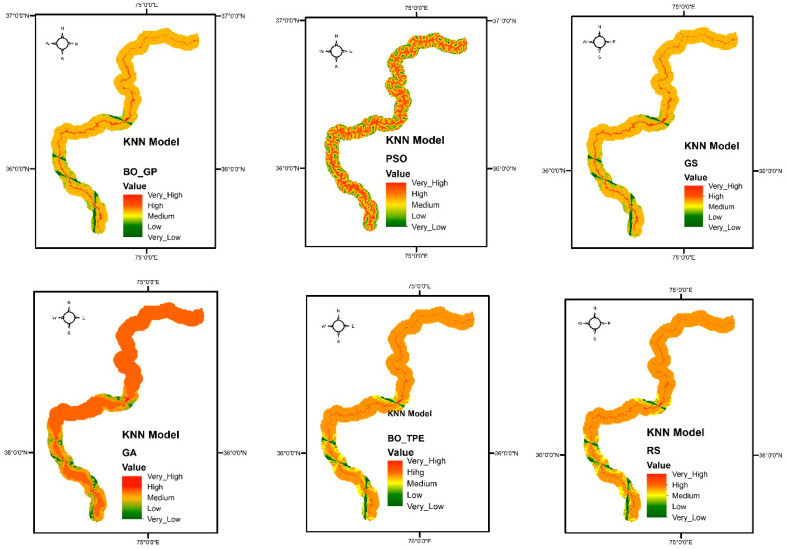
Landslide susceptibility maps obtained from K-nearest neighbors (KNN) model using six different optimization techniques: GS (Grid Search), RS (Random Search), BO-GP (Bayesian optimization Gaussian process), BO-TPE (Bayesian optimization Tree-structured Parzen estimator), GA (Genetic Algorithm), and PSO (Particle Swarm Optimization).

Discrete hyperparameters in KNN, like the number of neighbors to take into consideration, or k, are the main hyperparameters that require tuning. As explained in the section on hyperparameters, Bayesian optimization is the best choice in these conditions. As expected, the Bayesian approaches performed exceptionally well. For the KNN model, BO-TPE improved accuracy from the baseline algorithms RS and GS by 1% and 11%, respectively, while BO-GP improved results from RS and GS by 2% and 12%, respectively. The metaheuristic algorithms PSO and GA both performed similarly to BO-TPE and random search (RS), respectively see (Figure 6). The susceptibility maps created by KNN are shown in Figure 7.

#### 7.1.3. SVM

**Figure 8:**
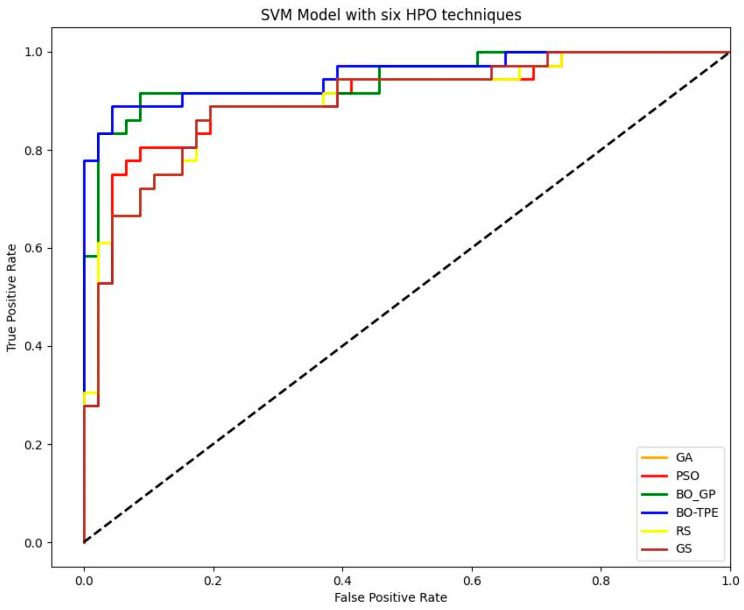
Receiver-operating characteristic (ROC) curve and AUC curve of support vector machine (SVM) model with GS (Grid Search), RS (Random Search), BO-GP (Bayesian optimization Gaussian process), BO-TPE (Bayesian optimization Tree-structured Parzen estimator), GA (Genetic Algorithm), and PSO (Particle Swarm Optimization) as parameter optimization techniques.

**Figure 9:**
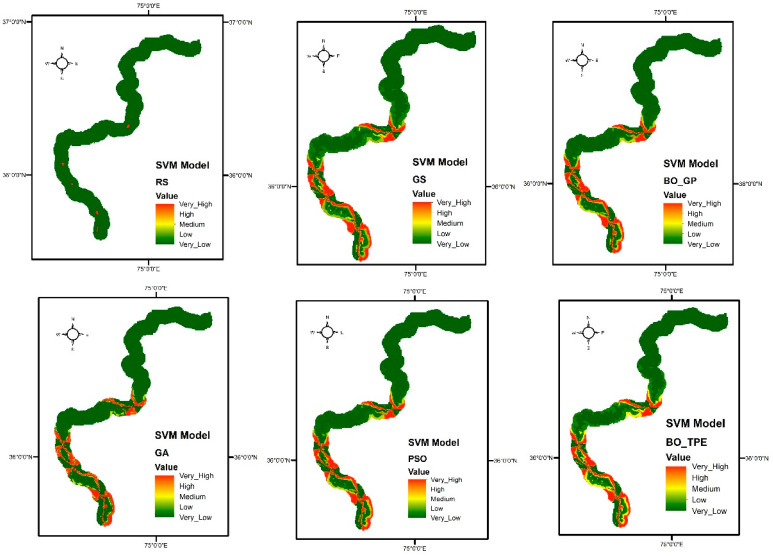
Landslide susceptibility maps obtained from Support Vector Machine (SVM) model using six different optimization techniques: GS (Grid Search), RS (Random Search), BO-GP (Bayesian optimization Gaussian process), BO-TPE (Bayesian optimization Tree-structure Parzen estimator), GA (Genetic Algorithm), and PSO (Particle Swarm Optimization).

Bayesian algorithms outperformed BO-TPE and produced 6% better outcomes than the baseline algorithms GS and RS with the SVM model, whereas BO-GP increased outcomes by 5%. PSO and GA both performed similarly, with results improving by 1%, as shown in the Figure 8. The susceptibility maps obtained through SVM classifier are shown in Figure 9.

It is evident from the obtained results that default hyperparameter configurations do not always yield the best model performance, emphasizing the significance of HPO techniques in improving model accuracy. Among the baseline models for HPO, Grid Search (GS) and Random Search (RS) were used. GS often had significantly higher computation time compared to other optimization techniques. RF and SVM models were faster than GS, but they did not guarantee finding near-optimal hyperparameter configurations. In contrast, Bayesian Optimization (BO) and multi-fidelity models consistently performed better in terms of accuracy. However, BO-GP required longer computation times due to its cubic time complexity.

Advantages and disadvantages based on our experimental results for the different hyperparameter optimization techniques can be summarized as follows. Grid Search is easy to implement, exhaustively searches the hyperparameter space, but is computationally expensive for large search spaces and may not find near-optimal configurations. Random Search is simple to implement, explores different hyperparameter combinations, but can be inefficient in finding optimal configurations, and randomness may lead to suboptimal results. Bayesian Optimization with Gaussian Process (BO-GP) is efficient in handling continuous hyperparameters, good at modeling complex relationships, but can be computationally intensive for large datasets or complex models. Bayesian Optimization with Tree-structured Parzen Estimator (BO-TPE) is efficient in handling conditional hyperparameters, performs well in small configuration spaces, but may require more evaluations to find optimal configurations in larger search spaces. Genetic Algorithm (GA) is suitable for large search spaces, can handle both continuous and discrete hyperparameters, but may take longer to converge, and the performance depends on the encoding and selection mechanisms. Particle Swarm Optimization (PSO) is efficient in parallel execution and suitable for models with vast configuration spaces, but performance may be sensitive to parameter settings, and it can converge to local optima.

For the RF classifier, PSO and GA outperformed other methods, while BO-TPE and BO-GP also achieved high accuracy. PSO’s parallel execution capability made it particularly effective for models with multiple hyperparameters of various types. GA performed well for models with large configuration spaces. BO-TPE excelled in handling conditional hyperparameters, while BO-GP performed well in smaller configuration spaces. For the SVM classifier, BO-TPE and BO-GP demonstrated superior accuracy compared to GS and RS. PSO and GA achieved similar results, while RS and GS performed relatively worse. BO-TPE’s ability to handle conditional hyperparameters contributed to its success in improving accuracy. As for the KNN classifier, Bayesian algorithms, BO-TPE, and BO-GP, they outperformed other methods, and PSO and GA achieved similar results. GS and RS had lower accuracy. Bayesian optimization proved to be effective for KNN’s discrete hyperparameter tuning.

## 8. Conclusions

Machine learning is now the go-to method for solving data-related issues and is extensively employed in many applications. The hyperparameters must be tweaked to fit particular datasets in order to use ML models to solve practical issues. However, the size of the created data is far larger in real life and manually adjusting hyperparameters requires a significant investment in computing power, so it is now imperative to optimize hyperparameters through an automated method. We have thoroughly covered the most recent findings in the field of hyperparameter optimization in this survey paper, as well as how to theoretically and practically apply them to various machine learning models. The hyperparameter types in an ML model are the primary consideration for choosing an HPO approach when applying optimization techniques to ML models. As a result, BO models are advised for small hyperparameter configuration spaces, while PSO is typically the ideal option for large configuration spaces. For ML data analysts, users, developers, and academics looking to apply and fine-tune ML models using appropriate HPO approaches and frameworks, we hope that our study will be a useful resource.

In recent years, the most advanced and innovative techniques used to solve optimization problems are metaheuristic algorithms and general-purpose optimization algorithms that are capable of finding near-optimal solutions for complex problems. Examples include Genetic Algorithms, Particle Swarm Optimization, and Ant Colony Optimization. These algorithms are inspired by natural processes and use iterative techniques to explore the search space efficiently. Deep learning techniques, particularly neural networks, have been applied to optimization problems in recent years. These methods leverage the power of neural networks to learn complex mappings between inputs and outputs and optimize objective functions directly [84]. Examples include Neural Architecture Search and Differentiable Programming. Bayesian Optimization is a sequential model-based optimization technique that efficiently searches for the optimum by building a probabilistic surrogate model of the objective function. It uses Bayesian inference to update the model as it explores the search space and focuses the search on promising regions. Convex optimization focuses on finding the global minimum of a convex objective function subject to a set of convex constraints. Although not new, advancements in convex optimization algorithms and software libraries have made solving large-scale convex optimization problems more practical and efficient [85]. Quantum optimization, also known as quantum computing optimization, is an emerging field that leverages the principles of quantum mechanics to solve optimization problems. Quantum algorithms, such as the Quantum Approximate Optimization Algorithm (QAOA) and Quantum Annealing, are being explored to tackle combinatorial optimization problems with large search spaces [86]. The field of optimization is continuously evolving, and new methods and techniques are being developed regularly. Therefore, the cutting-edge methods may vary over time as new advancements are made.

## Figures and Tables

**Table 1:** Hyperparameters of evaluated ML models’ configuration space.

ML Model	Hyperparameter	Type	Search Space
RF Classifier	n_estimators	Discrete	[10, 100]
	max_depth	Discrete	[5, 50]
	min_samples_split	Discrete	[2, 11]
	min_samples_leaf	Discrete	[1, 11]
	criterion	Categorical	[’gini’, ’entropy’]
	max_features	Discrete	[1, 64]
SVM Classifier	C	Continuous	[0.1, 50]
	Kernel	Categorical	[’linear’, ’poly’, ’rbf’, ’sigmoid’]
KNN Classifier	n_neighbors	Discrete	[1, 20]

**Table 2:** Summary of the landslide conditioning variable used in our case study.

Factors	Classes	Class Percentage %	Landslide Percentage %	Reclassification
Slope (°)	Very Gentle Slope < 5°	17.36	21.11	Geometrical interval reclassification
	Gentle Slope 5–15°	20.87	28.37	
	Moderately Steep Slope 15–30°	26.64	37.89	
	Steep Slope 30–45°	24.40	10.90	
	Escarpments > 45°	10.71	1.73	
Aspect	Flat (−1)	22.86	7.04	Remained unmodified (as in source data)
	North (0–22)	21.47	7.03	
	Northeast (22–67)	14.85	5.00	
	East (67–112)	8.00	11.86	
	Southeast (112–157)	5.22	14.3	
	South (157–202)	2.84	14.40	
	Southwest (202–247)	6.46	12.41	
	West (247–292)	7.19	16.03	
	Northwest (292–337)	11.07	11.96	
Land Cover	Dense Conifer	0.38	12.73	
	Sparse Conifer	0.25	12.80	
	Broadleaved, Conifer	1.52	10.86	
	Grasses/Shrubs	25.54	10.3	
	Agriculture Land	5.78	10.40	
	Soil/Rocks	56.55	14.51	
	Snow/Glacier	8.89	12.03	
	Water	1.06	16.96	
Geology	Cretaceous sandstone	13.70	6.38	
	Devonian–Carboniferous	12.34	5.80	
	Chalt Group	1.43	8.43	
	Hunza plutonic unit	4.74	10.74	
	Paragneisses	11.38	11.34	
	Yasin group	10.80	10.70	
	Gilgit complex	5.80	9.58	
	Trondhjemite	15.65	9.32	
	Permian massive limestone	6.51	6.61	
	Permanent ice	12.61	3.51	
	Quaternary alluvium	0.32	8.65	
	Triassic massive limestone and dolomite	1.58	7.80	
	snow	3.08	2.00	
Proximity to Stream (meter)	0–100 m	19.37	18.52	Geometrical interval reclassification
	100–200	10.26	21.63	
	200–300	10.78	25.16	
	300–400	13.95	26.12	
	400–500	18.69	6.23	
	>500	26.92	2.34	
Proximity to Road (meter)	0–100 m	81.08	25.70	
	100–200	10.34	25.19	
	200–300	6.72	27.09	
	300–400	1.25	12.02	
	400–500	0.60	10.00	
Proximity to Fault (meter)	000–1000 m	29.76	27.30	
	2000–3000	36.25	37.40	
	>3000	34.15	35.03	

**Table 3:** Comparison of popular HPO algorithms (where *n* denotes the number of values for the hyperparameters and *k* the number of hyperparameters).

HPO Method	Strengths	Limitations	Time Complexity
GS	Straightforward	Inefficient without categorical HPs and time-consuming.	O($n^{k}$)
RS	It is more effective than GS and supports parallelism.	Does not take into account prior outcomes. Ineffective when used with conditional HPs.	*O*(*n*)
BO-GP	For continuous HPs, fast convergence speed.	Poor parallelization ability; ineffective with conditional HPs.	$O\left(n^{3}\right)$
BO-TPE	Effective with all HP types. Maintains conditional dependencies.	Poor parallelization ability.	$O\left(nlogn\right)$
GA	All HPs are effective with it, and it does not need excellent initialization.	Poor parallelization ability.	$O\left(n^{2}\right)$
PSO	Enables parallelization; is effective with all sorts of HPs.	Needs to be initialized properly.	$O\left(nlogn\right)$

**Table 4:** Performance analysis of the RF classifier using HPO methods on the landslide dataset.

Optimization Algorithm	Accuracy (%)	CT(s)
GS	0.90730	4.70
RS	0.92663	3.91
BO-GP	0.93266	16.94
BO-TPE	0.94112	1.43
GA	0.94957	4.90
PSO	0.95923	3.12

**Table 5:** Performance analysis of the SVM classifier using HPO methods on the landslide dataset.

Optimization Algorithm	Accuracy (%)	CT(s)
BO-TPE	0.95289	0.55
BO-GP	0.94565	5.78
PSO	0.90277	0.43
GA	0.90277	1.18
RS	0.89855	0.73
GS	0.89794	1.23

**Table 6:** Performance analysis of the KNN classifier using HPO methods on the landslide dataset.

Optimization Algorithm	Accuracy (%)	CT(s)
BO-GP	0.90247	1.21
BO-TPE	0.89462	2.23
PSO	0.89462	1.65
GA	0.88194	2.43
RS	0.88194	6.41
GS	0.78925	7.68

## Data Availability

The data presented in the study are available on request from the first and corresponding author. The data are not publicly available due to the thesis that is being prepared from these data.

## References

[1] Polanco C. (2014). Add a new comment. Science.

[2] Zöller M.-A., Huber M.F. (2021). Benchmark and survey of automated machine learning frameworks. J. Artif. Intell. Res..

[3] Elshawi R., Maher M., Sakr S. (2019). Automated machine learning: State-of-the-art and open challenges. arXiv.

[4] DeCastro-García N., Munoz Castaneda A.L., Escudero Garcia D., Carriegos M.V. (2019). Effect of the sampling of a dataset in the hyperparameter optimization phase over the efficiency of a machine learning algorithm. Complexity.

[5] Abreu S. (2019). Automated architecture design for deep neural networks. arXiv.

[6] Olof S.S. (2018). A Comparative Study of Black-Box Optimization Algorithms for Tuning of Hyper-Parameters in Deep Neural Networks.

[7] Luo G. (2016). A review of automatic selection methods for machine learning algorithms and hyper-parameter values. Netw. Model. Anal. Health Inform. Bioinform..

[8] Maclaurin D., Duvenaud D., Adams R. Gradient-based hyperparameter optimization through reversible learning. Proceedings of the International Conference on Machine Learning.

[9] Bergstra J., Bardenet R., Bengio Y., Kégl B. (2011). Algorithms for hyper-parameter optimization. Advances in Neural Information Processing Systems.

[10] Bergstra J., Bengio Y. (2012). Random search for hyper-parameter optimization. J. Mach. Learn. Res..

[11] Eggensperger K., Feurer M., Hutter F., Bergstra J., Snoek J., Hoos H., Leyton-Brown K. Towards an empirical foundation for assessing bayesian optimization of hyperparameters. Proceedings of the NIPS Workshop on Bayesian Optimization in Theory and Practice.

[12] Eggensperger K., Hutter F., Hoos H., Leyton-Brown K. Efficient benchmarking of hyperparameter optimizers via surrogates. Proceedings of the AAAI Conference on Artificial Intelligence.

[13] Li L., Jamieson K., DeSalvo G., Rostamizadeh A., Talwalkar A. (2017). Hyperband: A novel bandit-based approach to hyperparameter optimization. J. Mach. Learn. Res..

[14] Yao Q., Wang M., Chen Y., Dai W., Li Y.-F., Tu W.-W., Yang Q., Yu Y. (2018). Taking human out of learning applications: A survey on automated machine learning. arXiv.

[15] Lessmann S., Stahlbock R., Crone S.F. Optimizing hyperparameters of support vector machines by genetic algorithms. Proceedings of the IC-AI.

[16] Lorenzo P.R., Nalepa J., Kawulok M., Ramos L.S., Pastor J.R. Particle swarm optimization for hyper-parameter selection in deep neural networks. Proceedings of the Genetic and Evolutionary Computation Conference.

[17] Li H., Chaudhari P., Yang H., Lam M., Ravichandran A., Bhotika R., Soatto S. (2020). Rethinking the hyperparameters for fine-tuning. arXiv.

[18] Poojary R., Raina R., Mondal A.K. (2021). Effect of data-augmentation on fine-tuned CNN model performance. IAES Int. J. Artif. Intell..

[19] Cattan Y., Choquette-Choo C.A., Papernot N., Thakurta A. (2022). Fine-tuning with differential privacy necessitates an additional hyperparameter search. arXiv.

[20] Ahmad Z., Li J., Mahmood T. (2023). Adaptive Hyperparameter Fine-Tuning for Boosting the Robustness and Quality of the Particle Swarm Optimization Algorithm for Non-Linear RBF Neural Network Modelling and Its Applications. Mathematics.

[21] Shen X., Plested J., Caldwell S., Zhong Y., Gedeon T. (2022). AMF: Adaptable Weighting Fusion with Multiple Fine-tuning for Image Classification. arXiv.

[22] Iqbal J., Ali M., Ali A., Raza D., Bashir F., Ali F., Hussain S., Afzal Z. (2020). Investigation of cryosphere dynamics variations in the upper indus basin using remote sensing and gis. Int. Arch. Photogramm. Remote Sens. Spat. Inf. Sci..

[23] Jamil A., Khan A.A., Bayram B., Iqbal J., Amin G., Yesiltepe M., Hussain D. Spatio-temporal glacier change detection using deep learning: A case study of Shishper Glacier in Hunza. Proceedings of the International Symposium on Applied Geoinformatics.

[24] Watanabe S., Hutter F. (2022). c-TPE: Generalizing tree-structured Parzen estimator with inequality constraints for continuous and categorical hyperparameter optimization. arXiv.

[25] Yang L., Shami A. (2020). On hyperparameter optimization of machine learning algorithms: Theory and practice. Neurocomputing.

[26] Zhao M., Li J. Tuning the hyper-parameters of CMA-ES with tree-structured Parzen estimators. Proceedings of the 2018 Tenth International Conference on Advanced Computational Intelligence (ICACI).

[27] Kelkar K.M., Bakal J. Hyper parameter tuning of random forest algorithm for affective learning system. Proceedings of the 2020 Third International Conference on Smart Systems and Inventive Technology (ICSSIT).

[28] Liu R., Liu E., Yang J., Li M., Wang F. Optimizing the hyper-parameters for SVM by combining evolution strategies with a grid search. Proceedings of the Intelligent Control and Automation: International Conference on Intelligent Computing, ICIC 2006.

[29] Kalita D.J., Singh V.P., Kumar V. (2020). A survey on SVM hyper-parameters optimization techniques. Social Networking and Computational Intelligence: Proceedings of SCI-2018, Bhopal, India, 5–6 October 2018.

[30] Polepaka S., Kumar R.R., Katukam S., Potluri S.V., Abburi S.D., Peddineni M., Islavath N., Anumandla M.R. Heart Disease Prediction-based on Conventional KNN and Tuned-Hyper Parameters of KNN: An Insight. Proceedings of the 2023 International Conference on Computer Communication and Informatics (ICCCI).

[31] Koutsoukas A., Monaghan K.J., Li X., Huan J. (2017). Deep-learning: Investigating deep neural networks hyper-parameters and comparison of performance to shallow methods for modeling bioactivity data. J. Cheminform..

[32] Ogilvie H.A., Heled J., Xie D., Drummond A.J. (2016). Computational performance and statistical accuracy of *BEAST and comparisons with other methods. Syst. Biol..

[33] Pritsker M. (1997). Evaluating value at risk methodologies: Accuracy versus computational time. J. Financ. Serv. Res..

[34] Pedregosa F., Varoquaux G., Gramfort A., Michel V., Thirion B., Grisel O., Blondel M., Prettenhofer P., Weiss R., Dubourg V. (2011). Scikit-learn: Machine learning in Python. J. Mach. Learn. Res..

[35] Head T., Louppe G., Shcherbatyi I., Schröder C., Campos N., MechCoder, fcharras, Zé Vinícius, cmmalone, nel215 (2018). scikit-optimize/scikit-optimize: v0.5.2. https://zenodo.org/record/1207017.

[36] Komer B., Bergstra J., Eliasmith C. (2014). Hyperopt-sklearn: Automatic hyperparameter configuration for scikit-learn. ICML Workshop on AutoML.

[37] Claesen M., Simm J., Popovic D., Moreau Y., De Moor B. (2014). Easy hyperparameter search using optunity. arXiv.

[38] Falkner S., Klein A., Hutter F. BOHB: Robust and efficient hyperparameter optimization at scale. Proceedings of the International Conference on Machine Learning.

[39] Olson R.S., Moore J.H. TPOT: A tree-based pipeline optimization tool for automating machine learning. Proceedings of the Workshop on Automatic Machine Learning.

[40] Dhuime B., Bosch D., Garrido C.J., Bodinier J.-L., Bruguier O., Hussain S.S., Dawood H. (2009). Geochemical architecture of the lower-to middle-crustal section of a paleo-island arc (Kohistan Complex, Jijal–Kamila area, northern Pakistan): Implications for the evolution of an oceanic subduction zone. J. Petrol..

[41] Rahman N.U., Song H., Benzhong X., Rehman S.U., Rehman G., Majid A., Iqbal J., Hussain G. (2022). Middle-Late Permian and Early Triassic foraminiferal assemblages in the Western Salt Range, Pakistan. Rud. -Geološko-Naft. Zb..

[42] Baloch M.Y.J., Zhang W., Al Shoumik B.A., Nigar A., Elhassan A.A., Elshekh A.E., Bashir M.O., Ebrahim A.F.M.S., Iqbal J. (2022). Hydrogeochemical mechanism associated with land use land cover indices using geospatial, remote sensing techniques, and health risks model. Sustainability.

[43] Iqbal J., Amin G., Su C., Haroon E., Baloch M.Y.J. (2023). Assessment of Landcover Impacts on the Groundwater Quality Using Hydrogeochemical and Geospatial Techniques. https://www.researchsquare.com/article/rs-2771650/v1.

[44] Tong Y., Yu B. (2022). Research on hyper-parameter optimization of activity recognition algorithm based on improved cuckoo search. Entropy.

[45] Sun X., Lin J., Bischl B. ReinBo: Machine learning pipeline conditional hierarchy search and configuration with Bayesian optimization embedded reinforcement learning. Proceedings of the Machine Learning and Knowledge Discovery in Databases: International Workshops of ECML PKDD 2019.

[46] Nguyen D., Gupta S., Rana S., Shilton A., Venkatesh S. Bayesian optimization for categorical and category-specific continuous inputs. Proceedings of the AAAI Conference on Artificial Intelligence.

[47] Ilievski I., Akhtar T., Feng J., Shoemaker C. Efficient hyperparameter optimization for deep learning algorithms using deterministic RBF surrogates. Proceedings of the AAAI Conference on Artificial Intelligence.

[48] Witt C. Worst-case and average-case approximations by simple randomized search heuristics. Proceedings of the STACS 2005: 22nd Annual Symposium on Theoretical Aspects of Computer Science.

[49] Hutter F., Kotthoff L., Vanschoren J. (2019). Automated Machine Learning: Methods, Systems, Challenges.

[50] Nguyen V. Bayesian optimization for accelerating hyper-parameter tuning. Proceedings of the 2019 IEEE Second International Conference on Artificial Intelligence and Knowledge Engineering (AIKE).

[51] Sanders S., Giraud-Carrier C. Informing the use of hyperparameter optimization through metalearning. Proceedings of the 2017 IEEE International Conference on Data Mining (ICDM).

[52] Hazan E., Klivans A., Yuan Y. (2017). Hyperparameter optimization: A spectral approach. arXiv.

[53] Hutter F., Hoos H.H., Leyton-Brown K. Sequential model-based optimization for general algorithm configuration. Proceedings of the Learning and Intelligent Optimization: 5th International Conference, LION 5.

[54] Dewancker I., McCourt M., Clark S. (2015). Bayesian Optimization Primer. https://static.sigopt.com/b/20a144d208ef255d3b981ce419667ec25d8412e2/static/pdf/SigOpt_Bayesian_Optimization_Primer.pdf.

[55] Gogna A., Tayal A. (2013). Metaheuristics: Review and application. J. Exp. Theor. Artif. Intell..

[56] Itano F., de Sousa M.A.d.A., Del-Moral-Hernandez E. Extending MLP ANN hyper-parameters Optimization by using Genetic Algorithm. Proceedings of the 2018 International Joint Conference on Neural Networks (IJCNN).

[57] Kazimipour B., Li X., Qin A.K. A review of population initialization techniques for evolutionary algorithms. Proceedings of the 2014 IEEE Congress on Evolutionary Computation (CEC).

[58] Rahnamayan S., Tizhoosh H.R., Salama M.M. (2007). A novel population initialization method for accelerating evolutionary algorithms. Comput. Math. Appl..

[59] Lobo F.G., Goldberg D.E., Pelikan M. Time complexity of genetic algorithms on exponentially scaled problems. Proceedings of the 2nd Annual Conference on Genetic and Evolutionary Computation.

[60] Shi Y., Eberhart R.C. Parameter selection in particle swarm optimization. Proceedings of the Evolutionary Programming VII: 7th International Conference, EP98.

[61] Yan X.-H., He F.-Z., Chen Y.-L. (2017). 基于野草扰动粒子群算法的新型软硬件划分方法. 计算机科学技术学报.

[62] Min-Yuan C., Kuo-Yu H., Merciawati H. (2018). Multiobjective Dynamic-Guiding PSO for Optimizing Work Shift Schedules. J. Constr. Eng. Manag..

[63] Wang H., Wu Z., Wang J., Dong X., Yu S., Chen C. A new population initialization method based on space transformation search. Proceedings of the 2009 Fifth International Conference on Natural Computation.

[64] Sun S., Cao Z., Zhu H., Zhao J. (2019). A survey of optimization methods from a machine learning perspective. IEEE Trans. Cybern..

[65] McCarl B.A., Spreen T.H. (1997). Applied Mathematical Programming Using Algebraic Systems.

[66] Bubeck S. (2015). Konvex optimering: Algoritmer och komplexitet. Found. Trends^®^ Mach. Learn..

[67] Abbas F., Zhang F., Iqbal J., Abbas F., Alrefaei A.F., Albeshr M. (2023). Assessing the Dimensionality Reduction of the Geospatial Dataset Using Principal Component Analysis (PCA) and Its Impact on the Accuracy and Performance of Ensembled and Non-ensembled Algorithms. Preprints.

[68] Abbas F., Zhang F., Abbas F., Ismail M., Iqbal J., Hussain D., Khan G., Alrefaei A.F., Albeshr M.F. (2023). Landslide Susceptibility Mapping: Analysis of Different Feature Selection Techniques with Artificial Neural Network Tuned by Bayesian and Metaheuristic Algorithms. Preprints.

[69] Shahriari B., Bouchard-Côté A., Freitas N. Unbounded Bayesian optimization via regularization. Proceedings of the Artificial Intelligence and Statistics.

[70] Diaz G.I., Fokoue-Nkoutche A., Nannicini G., Samulowitz H. (2017). An effective algorithm for hyperparameter optimization of neural networks. IBM J. Res. Dev..

[71] Gambella C., Ghaddar B., Naoum-Sawaya J. (2021). Optimization problems for machine learning: A survey. Eur. J. Oper. Res..

[72] Sparks E.R., Talwalkar A., Haas D., Franklin M.J., Jordan M.I., Kraska T. Automating model search for large scale machine learning. Proceedings of the Sixth ACM Symposium on Cloud Computing.

[73] Nocedal J., Wright S.J. (1999). Numerical Optimization.

[74] Chen C., Yan C., Li Y. (2015). A robust weighted least squares support vector regression based on least trimmed squares. Neurocomputing.

[75] Yang L., Muresan R., Al-Dweik A., Hadjileontiadis L.J. (2018). Image-based visibility estimation algorithm for intelligent transportation systems. IEEE Access.

[76] Zhang J., Jin R., Yang Y., Hauptmann A. Modified logistic regression: An approximation to SVM and its applications in large-scale text categorization. Proceedings of the Twentieth International Conference on Machine Learning (ICML-2003).

[77] Soliman O.S., Mahmoud A.S. A classification system for remote sensing satellite images using support vector machine with non-linear kernel functions. Proceedings of the 2012 8th International Conference on Informatics and Systems (INFOS).

[78] Safavian S.R., Landgrebe D. (1991). A survey of decision tree classifier methodology. IEEE Trans. Syst. Man Cybern..

[79] Manias D.M., Jammal M., Hawilo H., Shami A., Heidari P., Larabi A., Brunner R. Machine learning for performance-aware virtual network function placement. Proceedings of the 2019 IEEE Global Communications Conference (GLOBECOM).

[80] Yang L., Moubayed A., Hamieh I., Shami A. Tree-based intelligent intrusion detection system in internet of vehicles. Proceedings of the 2019 IEEE Global Communications Conference (GLOBECOM).

[81] Injadat M., Salo F., Nassif A.B., Essex A., Shami A. Bayesian optimization with machine learning algorithms towards anomaly detection. Proceedings of the 2018 IEEE Global Communications Conference (GLOBECOM).

[82] Arjunan K., Modi C.N. An enhanced intrusion detection framework for securing network layer of cloud computing. Proceedings of the 2017 ISEA Asia Security and Privacy (ISEASP).

[83] Dietterich T.G. Ensemble methods in machine learning. Proceedings of the Multiple Classifier Systems: First International Workshop, MCS 2000.

[84] Ning C., You F. (2019). Optimization under uncertainty in the era of big data and deep learning: When machine learning meets mathematical programming. Comput. Chem. Eng..

[85] Boyd S.P., Vandenberghe L. (2004). Convex Optimization.

[86] Hogg T., Portnov D. (2000). Quantum optimization. Inf. Sci..

